# Correction of postpneumonectomy syndrome after bronchopleural fistula

**DOI:** 10.1186/s13019-019-0897-8

**Published:** 2019-04-08

**Authors:** Max S. Yudovich, Eliza W. Beal, Desmond M. D’Souza, Susan D. Moffatt-Bruce, Robert E. Merritt, Peter J. Kneuertz

**Affiliations:** Thoracic Surgery Division, Department of Surgery, The Ohio State University Wexner Medical Center, Doan Hall N846, 410 West 10th Avenue, Columbus, OH 43210 USA

**Keywords:** Postpneumonectomy syndrome, Bronchopleural fistula, Pneumonectomy complication

## Abstract

**Background:**

Postpneumonectomy syndrome is a rare complication of pneumonectomy characterized by mediastinal shift toward the pneumonectomy cavity. Bronchopleural fistula (BPF) is another infrequent complication causing infection of the pneumonectomy space. The combination of both complications poses a major clinical challenge.

**Case presentation:**

We present a case of successful surgical correction of postpneumonectomy syndrome in a patient with previous BPF and associated empyema. Intraoperative gram stain and cultures were used to rule out a persistent infection. Medialization of the mid and lower mediastinum was performed avoiding manipulation of the bronchial stump and its muscle buttress following previous BPF closure. Placement of intrathoracic implants resulted in resolution of symptoms.

**Conclusions:**

This case highlights important clinical considerations for correction of a postpneumonectomy syndrome following BPF. A subclinical infection should be ruled out prior to placement of implants. Partial medialization and symptomatic improvement may be accomplished without disrupting the bronchial stump after healed BPF.

## Background

Postpneumonectomy syndrome is a complication of pneumonectomy characterized by airway obstruction, pulmonary hypertension, and esophageal compression due to mediastinal shift toward the pneumonectomy cavity. This condition is most typically caused by a right pneumonectomy and the resulting rightward and posterior shift of the mediastinum, but cases of postpneumonectomy syndrome following left pneumonectomy have also been reported [1, 2]. Patients with postpneumonectomy syndrome often complain of dyspnea on exertion, cough, and stridor as a result of compression of the tracheobronchial tree between the pulmonary artery and descending aorta [3]. Dysphagia may also be present due to compression of the esophagus between the inferior vena cava and descending aorta [4].

Postpneumonectomy syndrome can be surgically corrected by placement of saline prosthetic implants to counter the mediastinal shift [1–6]. More complex surgical corrections including pericardial fixation, tracheobronchial reconstruction, and vertebral body resection have also been reported [5]. Rarely, postpneumonectomy syndrome may present following bronchopleural fistula (BPF), which provides additional unique clinical challenges.

## Case presentation

A 51-year-old female with a history of rheumatoid arthritis and a 10.5-pack-year smoking history presented with an aspergilloma in her right lung. After failing medical management, she was treated with a right pneumonectomy at an outside institution. This was complicated by BPF and empyema of the pneumonectomy cavity. She underwent two additional thoracotomies requiring rib resection, and placement of serratus anterior and later latissimus dorsi flap to close the fistula. Seven months following her last operation, she presented to us with stridor, persistent cough, and dysphagia, concerning for postpneumonectomy syndrome. Review of last computed tomography (CT) imaging from three months after the pneumonectomy revealed a multiloculated pleural space, with air fluid levels in the pneumonectomy cavity. An updated CT scan showed interval progressive rightward mediastinal shift with nearly complete obliteration of the pneumonectomy cavity by the heart (Fig. 1). A bronchoscopy was performed, which demonstrated narrowing of the left mainstem bronchus (Fig. 2a) and stenosis of the lower lobe bronchial orifice due to external compression of the airways. Results of a previous complex right-sided BPF with two areas of disrupted bronchial staple line were noted (Fig. 2b).

**Fig. 1:**
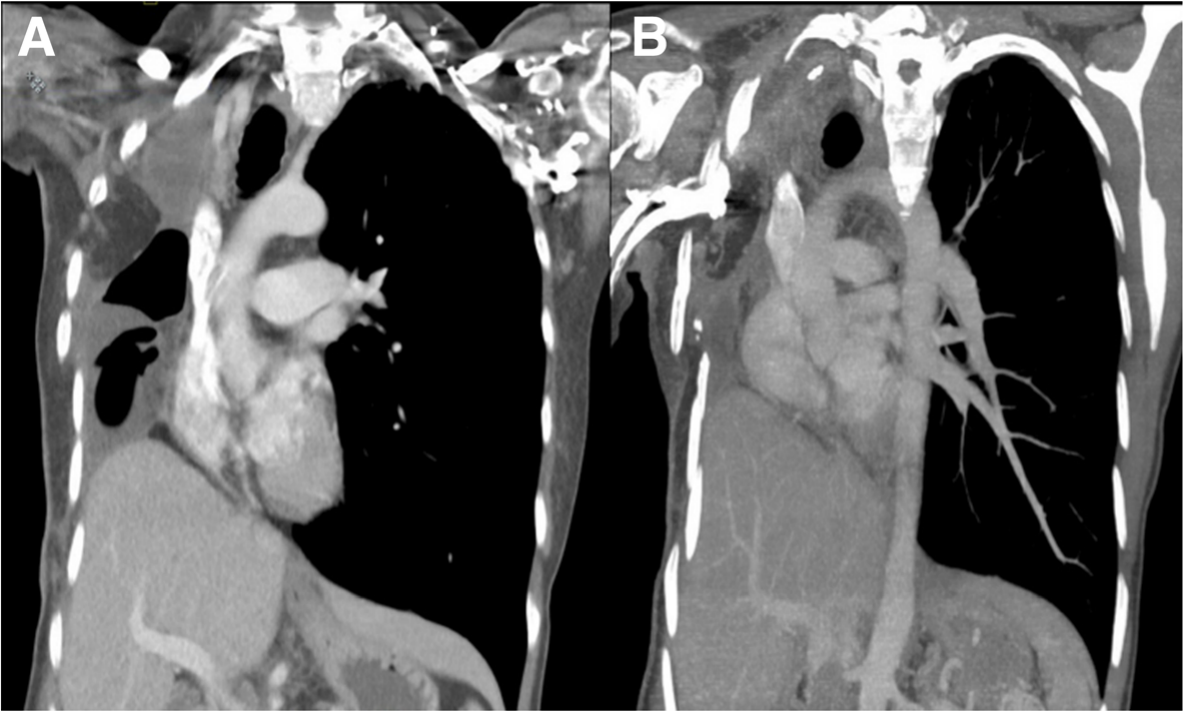
CT scans prior to postpneumonectomy syndrome correction: **a** initially showing multiloculated air-fluid levels three months after right pneumonectomy shortly after closure of a bronchopleural fistula, and **b** progressive rightward mediastinal shift with obliteration and resolution of the loculations

**Fig. 2:**
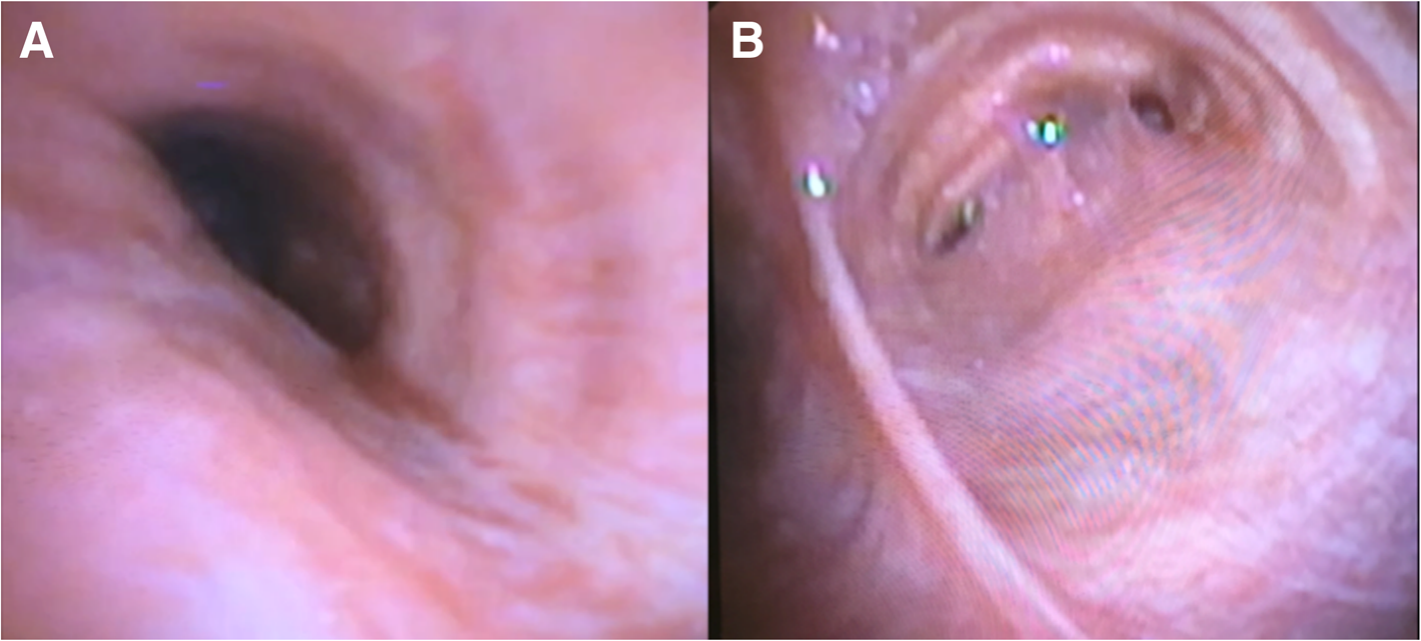
**a** Preoperative bronchoscopy image showing external compression of the left mainstem bronchus. **b** Bronchoscopy image showing evidence of previous complex bronchopleural fistulae in the right mainstem bronchus

The patient elected to proceed with operative correction of her postpneumonectomy syndrome. A thoracotomy in the fifth intercostal space was performed and dense adhesions in the chest with rotation of mediastinal structures were faced. Upon entering the pleural space, a small loculated serous fluid collection was encountered. To rule out an infected field, the pleural rind and fluid samples were sent for intraoperative gram stains, which returned negative. Cultures were also submitted. The mediastinum was mobilized from the chest wall, taking care to avoid damage to the muscle flaps, which had previously sealed the BPF. As a result, only the mid and inferior portion of the mediastinum was mobilized. A saline immersion test was performed to ensure the integrity of the muscle flap seal over the right mainstem bronchus. The implants were sized based on measuring the amount of saline instilled in the chest, and close hemodynamic monitoring of arterial and central venous pressures. Before placing the implants, the thoracotomy was closed temporarily after placement of implant sizers, monitoring hemodynamics to ensure there was no right heart compression. Two implants (250 mL and 100 mL) were placed into the pleural cavity, and the thoracotomy was closed. The postoperative recovery was uneventful. The patient was discharged on post-operative day 5. She noted complete resolution of her stridor, cough, as well as dysphagia four weeks post-operatively. Her post-operative chest radiograph showed partial medialization of the inferior mediastinum with persistent rightward deviation of the proximal trachea (Fig. 3). At time preparation of this manuscript, the patient continues to have full resolution of symptoms at fourteen months following surgery.

**Fig. 3:**
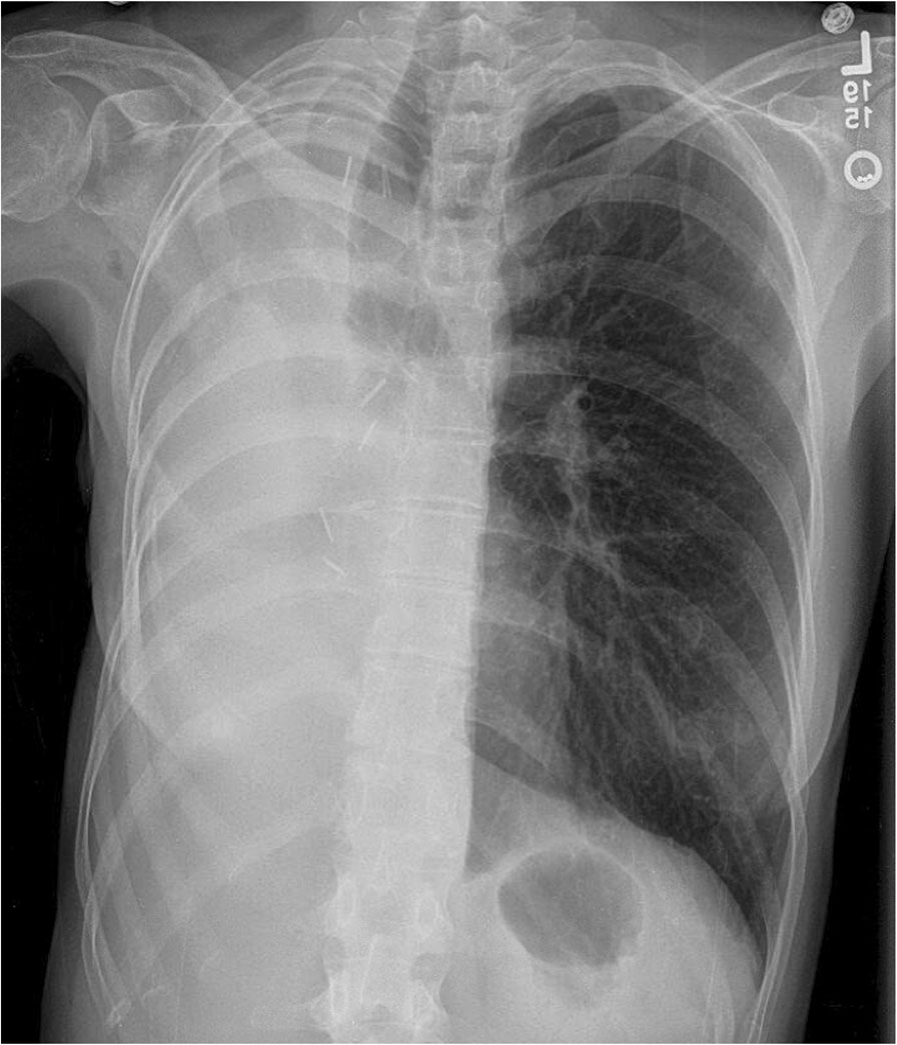
Chest radiograph four weeks after placement of saline implants shows medialization of the inferior mediastinum and residual rightward tracheal deviation

## Discussion and conclusions

We present a challenging case of a patient with postpneumonectomy syndrome in the setting of previous BPF following pneumonectomy for aspergilloma, which was successfully corrected with adhesiolysis and placement of implants. Reflecting on our experience with this case, there are two main problems, unique to the presentation of postpneumonectomy syndrome following BPF.

First, we had concern of a subclinical infection as a result of previous empyema. Even though the previous pneumonectomy pleural space had essentially obliterated, we found a small serous collection and fibrinous rind at time of operative exploration. Our approach was to check intraoperative gram stain to rule out residual colonization prior to introducing a foreign body. In the absence of organisms detected by intraoperative gram stain, we were assured to place the implants. The use of saline implants had been previously described for modified-thoracoplasty following aspergillosis-related lung resection [7]. Prophylactic antibiotics were continued for 24 h postoperatively, and negative cultures were confirmed. In the event of positive intra-operative gram stain, it would have been ill advised to introduce a foreign body at that time. The second concern was the ability to correct the mediastinal shift in a patient with previous bronchopleural fistula. A literature review of patients with postpneumonectomy syndrome showed a high success rate with use of saline implants. Of 55 patients treated with saline implants, 41 (75%) achieved symptom free outcomes over a median follow-up time of 2 years [5]. However, to our knowledge, correction of postpneumonectomy syndrome after BPF has not been previously described. As a result of the BPF, this patient had an excessive amount adhesions and scar tissue, as well bulky viable muscle flaps in the upper pleural space, which had closed the BPF. Previous case reports of postpneumonectomy syndrome describe the need for complete adhesiolysis of the mediastinum. To ensure optimal medialization of the mediastinum, it is typically recommended to divide scar tissue around the right bronchus and pneumonectomy stump [3]. For this patient, only the mid and inferior mediastinum was mobilized from the chest wall due to concern for disturbing the integrity of the existing muscle flap coverage over the healed BPF. The limited dissection allowed a relatively low volume of saline prosthesis to be implanted. Our patient received 350 mL of implants, whereas previous literature has calculated the median volume of saline between 805 and 945 mL [3, 5]. However, despite the partial mobilization of the mediastinum and limited volume of saline, the implants caused enough mediastinal correction to completely alleviate the patient’s symptoms.

In summary, postpneumonectomy syndrome may present in patients following bronchopleural fistula, and may be successfully treated with adhesiolysis and placement of saline implants. In this context, precautions should be taken to rule out subclinical infection or persistent empyema to ensure saline implant longevity and prevent postoperative complications. Sufficient medialization may be accomplished without extensive pneumonectomy stump dissection to achieve symptom resolution.

## Abbreviations

BPF: Bronchopleural fistula
CT: Computed tomography
